# Chromium – a scoping review for Nordic Nutrition Recommendations 2023

**DOI:** 10.29219/fnr.v67.10325

**Published:** 2023-12-06

**Authors:** Christine Henriksen, Susanne Bügel

**Affiliations:** 1 Department of Nutrition, Institute of Basic Medical Sciences, University of Oslo, Oslo, Norway; 2 Department of Nutrition, Exercise and Sports, Faculty of Science, University of Copenhagen, Frederiksberg C., Denmark

**Keywords:** chromium, trace elements, nutrition recommendations, toxicity

## Abstract

Trivalent chromium (CrIII) is the principal form of chromium found in diet and supplements. CrIII has been claimed to be involved in the regulation of carbohydrate, lipid, and protein metabolism. Hexavalent chromium (CrVI) is a carcinogen when inhaled, which is uncommon, and occurs mainly by occupational exposures. There is a concern about adverse health effects also from exposure to CrVI by contaminated drinking water, although data from human studies are limited. Chromium had no recommendation in the Nordic Nutrition Recommendations (NNR) 2012 and the European Food Safety Authority (EFSA) did not set any reference values either. Methods for evaluating chromium status are lacking, and there is still uncertainty about how chromium deficiency in humans manifests itself. The essentiality of chromium is also disputed. This scoping review revealed new research activity relating to high-dose chromium supplements and several health outcomes (overweight, obesity, and diabetes). Although these issues are related to health concerns in the Nordic or Baltic countries, the relevance for the NNR is modest, since such a high intake of chromium cannot be achieved by diet. Thus, no strong evidence was identified in the scientific literature that justifies a recommendation for chromium intake.

## Popular scientific summary

- Chromium is a trace element mainly in the form of trivalent chromium in foods and dietary supplements.
- Chromium is ubiquitously found in foods, but fish, whole grain products, nuts, pulses, spices, and processed meats are good sources.
- Data on dietary intakes in Nordic and Baltic countries is lacking.
- Whether chromium is an essential nutrient for humans is disputed.
- Chromium may be involved in the regulation of carbohydrate and lipid metabolism, but the evidence is limited.

In ionic form, chromium exists in many states. Trivalent chromium (CrIII) is the principal form of chromium found in foods and dietary supplements. It is ubiquitous in nature and occurs in the air, water, soil, and biological materials. CrIII may be involved in the regulation of carbohydrate and lipid metabolism, by enhancing insulin’s efficacy, but the evidence is limited. The essentiality of chromium is disputed, as no deficiencies are documented in healthy humans.

Hexavalent chromium (CrVI) forms chromates and dichromates that are strong oxidisers and can traverse biological membranes. CrVI compounds are used in industrial processes, including chrome pigment production, chrome plating, stainless steel manufacturing, and leather tanning. Fortunately, the emission of chromium to the environment has steadily declined in the Nordic countries during the last 20 years, but oral exposure to CrVI by drinking water may affect parts of the population. Although data from human studies are quite limited, there is a concern about adverse health effects also from oral exposure to CrVI. There is also an indication of contact allergy caused by chromium exposure.

The aim of this scoping review is to describe the totality of evidence for the role of chromium for health- related outcomes as a basis for setting and updating dietary reference values (DRVs) for the Nordic Nutrition Recommendations (NNR) 2023 (Box 1).

**Box 1. Background papers for Nordic Nutrition Recommendations 2023**

- This paper is one of many scoping reviews commissioned as part of the Nordic Nutrition Recommendations 2023 (NNR2023) project (1)
- The papers are included in the extended NNR2023 report but, for transparency, these scoping reviews are also published in Food & Nutrition Research
- The scoping reviews have been peer reviewed by independent experts in the research field according to the standard procedures of the journal
- The scoping reviews have also been subjected to public consultations (see report to be published by the NNR2023 project)
- The NNR2023 committee has served as the editorial board
- While these papers are a main fundament, the NNR2023 committee has the sole responsibility for setting dietary reference values in the NNR2023 project

## Methods

This scoping review follows the protocol developed within the NNR2023 project (1), and the sources of evidence used follow the eligibility criteria described by Christensen et al. (2). In the NNR2023 project, new scientific data and reasons for health concerns were first sought through a public consultation, which included scientists, authorities, and the general public in the Nordic and Baltic countries. Existing SRs were identified primarily by contacting major national food and health authorities and organisations, and a general web search. Chromium was not selected for a *de novo* systematic review and no qualified systematic review on chromium was identified by the NNR2023 project (3).

A literature search was conducted in MEDLINE, with the following search string inserted into PubMed, 14 March 2021: chromium[MeSH Terms] AND (“2011” [Date - Publication] : “3000”[Date - Publication]) AND review[Publication Type] AND Humans[Filter]. This search string picked up 146 publications, published later than 2011. Out of these, 58 articles were further scrutinised, and finally 28 articles were judged as relevant. Additionally, the search string did not pick up one publication (4). Some references from NNR2012 (published before 2012) were also included when relevant.

## Physiology

About 0.5% of the dietary intake of chromium is absorbed by the body via passive diffusion, and the remainder is excreted in the faeces. Generally, organic chromium compounds are absorbed two to 16 times better than inorganic forms, and chromium picolinate has higher bioavailability than chromium nicotinate (5). Simultaneous ascorbate administration increases chromium uptake in humans and animals, and chromium absorption is also higher in zinc- and iron-deficient animals. The element is transported in the circulation bound to albumin and transferrin, and is mainly excreted via urine. Urinary losses increase with metabolic stress and glucose intolerance can further drive this process. Only small amounts are eliminated in sweat and bile (6).

The exact biological function of chromium has not yet been determined. CrIII is considered to enhance insulin sensitivity, possibly through an influence on the glucose transporter 4 (GLUT-4) receptors (7). A low molecular weight chromium-binding substance is believed to be involved in the process (8). Furthermore, chromium inhibits hepatic enzyme 3-hydroxy-3-methyl-glutaryl-CoA reductase and affects cholesterol metabolism. It has also been suggested that chromium deficiency enhances inflammation, and influences carbohydrate-, lipid-, and protein metabolism indirectly via its effect on insulin action (9, 10)

In earlier animal studies, experimental chromium deficiency resulted in reduced glucose tolerance in spite of normal insulin levels. Other deficiency signs in animals included impaired growth, elevated levels of serum cholesterol and triglycerides, increased incidence of aortic plaques, corneal lesions, and decreased fertility and sperm count. More recent studies have however failed to reproduce symptoms of chromium deficiency in mammals (11).

CrVI forms are carcinogenic when inhaled. They are able to react directly with biological materials to produce reactive oxygen species (ROS), which are able to cause DNA damage and gene mutation. Besides causing mutations, CrVI promotes cancer development by stimulating angiogenesis through several signalling pathways (12).

## Assessment of nutrient status

There are no reliable biomarkers for chromium status. Blood and urine concentrations of chromium can be measured but are not good indicators because blood levels do not readily equilibrate with tissue stores. Plasma chromium levels may be reduced as a consequence of low intake, but are also reduced by acute illness. The lack of reliable biomarkers for chromium status, in combination with the absence of deficiency symptoms on low chromium diet, adds to the uncertainties about chromium being an essential trace element (13).

## Dietary intake in Nordic and Baltic countries

Analysis of chromium in foods requires special sampling procedures to avoid chromium contamination from the environment (air, stainless steel, etc.). Older analytical data on chromium contents in foods, produced before about 1980, should, therefore, be used with care. Fish, whole grain products, nuts, pulses, spices, and processed meats are good sources, but most other foods have at least low concentrations of chromium (<10 µg/100 g). Foods high in sugars, such as soft drinks and table sugar, are not only low in chromium content but also promote chromium loss (14). Studies analysing chromium intake in the diet of the Nordic countries are scarce, but estimated intakes in Swedish women was in the range of 20–160 µg/day in 1998 (15). The European Food Safety Authority (EFSA) has estimated the intake among adults in Europe to be 57–84 µg/day (4). No recent updates of intake data in the Nordic and Baltic countries were available for the NNR2023 revision. Many food supplements contain chromium in doses ranging from 50 to 100 µg per serving unit.

## Health outcomes relevant for Nordic and Baltic countries

### Deficiency

In the past, a handful of cases have been reported regarding possible chromium deficiency in humans after long-term parenteral nutrition. The symptoms observed were impaired glucose tolerance, weight loss, neuropathy, elevated concentrations of plasma fatty acids, depressed respiratory quotient, and abnormalities in nitrogen metabolism. The symptoms improved after chromium supplementation (200 µg/day). However, the reported concentrations of chromium in the blood and urine were above those considered normal even before the supplementation was initiated (16).

Trace elements, including chromium are now added to parenteral nutrition, and there have been reports of high chromium levels in blood and urine. This is of concern since toxic levels might have negative effect on kidney function, especially in infants (17).

### Obesity and other health outcomes

A number of chromium supplementation studies have investigated the effects of chromium supplements on weight, body composition, blood glucose, blood lipids, and markers of inflammation. Meta-analyses newer than 2012 are summarised in Table 1. In these studies, the dosage ranged from 50 to 1,000 µg and several CrIII-compounds were used such as chromium picolinate (organic), chromium nicotinate (organic), chromium chloride, as well as chromium yeast.

**Table 1:** Meta-analysis of chromium supplementation and health outcomes

Author	Methods	Population	Intervention	Results
Onakpoya 2013 (18)	Meta-analysis of 11 RCT	Overweight and obesity	137–1,000 µg chromium for 8–26 weeks	Significant reduction in weight (mean difference – 0.5 kg)
Tsang 2019 (19)	Meta-analysis of 20 RCT	Overweight and obesity	200–1,000 µg chromium for 16–24 weeks	Significant reduction in weight (mean difference – 0.75 kg)
Suksomboon 2014 (20)	Meta-analysis of 25 RCT	Type 2 diabetes	150–1,000 µg chromium for 4–24 weeks	Significant improved glycaemic control (mean difference for HbA1c – 0.55%, fasting glucose – 1.15 nmol)
Bailey 2014 (21)	Meta-analysis of 16 RCT	Type 2 diabetes and healthy individuals	200–1,000 µg chromium for 6–24 weeks	No significant effect
Asbaghi 2020 (22)	Meta-analysis of 28 RCT	Type 2 diabetes	50–1,000 µg chromium for 4–25 weeks	Significant improved glycaemic control (mean difference for HbA1c – 0.71%, fasting glucose – 19 mg/dL)
Zhang 2021 (24)	Meta-analysis of 28 RCT	Type 2 diabetes metabolic syndrome obesity polycystic ovary syndrome	200–1,000 µg chromium for 8–28 weeks	Significant improvement in CRP and TNF-α but not level of IL-6

A meta-analysis by Onakpoya et al. (18), including 11 randomised clinical trials (RCT), has explored the effect of chromium supplementation on weight in overweight and obese individuals. The results showed a statistically significant difference in weight loss favouring chromium over placebo (mean difference – 0.50 kg). In these studies, chromium supplements were given in dosages up to 1,000 µg, equivalent to > 12 times a normal daily intake.

A more recent meta-analyses by Tsang et al. (19) of 20 RCTs, confirmed that chromium supplementation was associated with significant improvements in weight (mean difference – 0.75 kg) and body composition (mean difference in body fat percentage – 0.68%) in subjects with obesity/overweight. This meta-analyses included some of the same RCTs as in Onakpoya et al. (18), and some additional publications. The effect size was medium and the clinical relevance of chromium as a weight loss aid remains uncertain. The relevance for the NNR is modest, since such high dosages cannot be achieved by the diet. Although the evidence from available RCTs shows that chromium supplementation leads to statistically significant reductions in body weight, the long-term effect is uncertain. The clinical relevance is also poor, as the magnitude of the weight loss was minimal (corresponding to <1% loss from baseline weight).

A meta-analysis by Suksomboon et al. (20) of 24 RCTs in patients with type 2 diabetes found that chromium supplementation significantly improved glycaemic control (mean difference for the 14 studies reporting on HbA1c – 0.55%, mean difference for the 24 studies reporting on fasting glucose – 1.15 mmol/L). Chromium supplementation also significantly reduced triglycerides and increased HDL-C levels in the 15 studies reporting on lipid profile. In contrast, Bailey et al. (21) did not find any significant effect of chromium supplementation in diabetic or healthy individuals. The conflicting results may reflect stricter methodology and different patient populations, as also studies with non-diabetic individuals were included in the latter meta-analysis. The most recent systematic review and meta-analysis of chromium supplementation in patients with type 2 diabetes included 28 RCTs. The results showed a significant reduction in all glycaemic control indices such as fasting glucose, insulin, and HbA1c after chromium supplementation (22). Although interventions of ≥12 weeks led to greater reduction of the mentioned indices compared to short time interventions, the long-term effect is uncertain. Again dosages up to 1,000 µg were given, which can only be achieved by dietary supplements.

Beneficial effects of chromium supplementation in patients with type 2 diabetes can be potentially explained by pharmacological rather than nutritional effects. One hypothesis is that chromium metabolism is altered in diabetes, because of increased urinary excretion of chromium due to poor renal function. Even though chromium appears to play a role in enhancing insulin action, further studies are required to understand the mechanism of chromium action in increasing insulin sensitivity and glucose uptake, with particular attention being turned to a potential role of transferrin and interactions with Fe(III) in Cr(III) transport, effects, and detoxification (7, 10, 23).

Zhang et al. (24) investigated the effect of chromium supplementation on inflammatory biomarkers (C-reactive protein (CRP), Tumor necrosis factor (TNF-α), and Interleukin (IL-6)) in type 2 diabetes, metabolicsyndrome, obesity, and polycystic ovary syndrome. Overall, the results of this systematic review and meta-analysis imply that chromium supplementation may help to improve some biomarkers of inflammation (CRP and TNF-α) but not the level of IL-6. However, a subgroup analysis showed that lower dosages (≤400 μg/day) and shorter duration of supplementation (≤12 week) were more effective in reducing CRP compared to higher dosage and longer duration of use. This raises the question as to whether an increased dosage or extended duration of supplement use may have a paradoxical effect. Another explanation may be that compliance to the supplements is reduced in long-term trials. More data from long-term follow-up studies are warranted.

In conclusion, there is no evidence of beneficial effects associated with increased chromium intake in healthy subjects. This is also in line with EFSA’s review of the topic (4). There seems to be a small, beneficial effect of high-dose chromium supplements on weight and glycaemic control in subjects who are overweight and have type 2 diabetes. Notably the long-term effect and the clinical relevance is uncertain, since other more efficient therapeutic approaches exists for these conditions. The relevance for the NNR is also modest since such high intake of chromium cannot be achieved by the diet in the Nordic or Baltic countries.

## Toxicity

CrIII has generally low toxicity, but mild adverse effects (nausea, watery stools, constipation, flatulence, vertigo, headaches, and urticaria) were observed in supplementation studies, at doses ranging from 150 to 1,000 µg/day. Due to the lack of adequate data, the EU Scientific Committee for Food (SCF) has not suggested a Tolerable Upper Intake Level (UL) for chromium (III) salts. The same conclusion was reached by the U.S. Food and Nutrition Board and the UK Expert Group on Vitamins and Minerals.

The consumption of chromium picolinate, a CrIII compound popular in many food supplements, has been debated because of possible carcinogenic effects. A scientific opinion from EFSA however, found chromium picolinate to be safe in doses up to 250 µg/day (25).

The carcinogenic potential of oral exposure to Cr(VI) in human is supported by several epidemiological and animal studies. Oral exposure normally results in a lower amount of Cr(VI) reaching into target cells compared to inhalation exposure, because Cr(VI) is reduced to Cr(III) in the gastrointestinal system. A portion of ingested Cr(VI) may however enter cells, and has the potential to initiate tumour formation. Therefore, Cr levels in drinking water must be set at levels that protect the population from chromium toxicity (26). The current drinking water standard established by the US Environmental Protection Agency (EPA) for total chromium is 0.1 mg/L or 100 ppb (27).

There have also been concerns about the effect of chromium (VI) on foetal development. However, a systematic review of eight studies did not detect any significant association between prenatal or foetal Cr (VI) exposures and adverse child outcome (premature birth, low birth weight, or impaired cognitive development) (28).

## Requirements and recommended intake

As described earlier, the role of chromium as an essential nutrient is still unclear. If chromium is an essential trace element, a deficiency should produce a disease or impairment of function. Methods for evaluating chromium status are lacking, and there is still uncertainty about how chromium deficiency in humans is manifested. Furthermore, there is no evidence of beneficial effects associated with increased chromium intake in healthy subjects.

SCF stated that ‘since data on the essentiality and metabolism of chromium are so sparse, the Committee is unable to specify any requirements’ (29). In 1991, the UK Committee on Medical Aspects of Food Policy, however, used balance studies and regression equations to calculate a theoretical requirement for adults of 23 µg/day, and stated that a safe and adequate intake level is believed to be greater than 25 µg/day for adults (30). However, no recommendation for intake were provided in the 2016 update (31). The U.S. Food and Nutrition Board estimated adequate intakes (AI) for chromium for different age groups based on calculations of well-balanced diets. For adults aged 19–50 years, the AI was estimated to be 35 µg/day for men and 25 µg/day for women (32). Despite these estimates, EFSA in 2014 concluded that no average requirement or dietary reference values for chromium could be defined (4). The conclusion was based on a systematic review including several relevant studies published between 1990 and 2011.

The NNR2012 did not include recommendations for chromium intake (33). For the present revision, new relevant publications from 2011 to 2021 were reviewed, and it is still impossible to establish requirements.

Data are also lacking on the requirements for chromium during pregnancy, but the U.S. Food and Nutrition Board suggests an increase of 5 µg/day during pregnancy over the usual chromium intake (34). Within Europe, mean chromium concentrations in human breast milk range between 0.14 and 10.8 μg/L , and the chromium concentration appears to be independent of maternal chromium intake (4). EFSA did not recommend any chromium intake for pregnant or lactating women (4).

### Data gaps for future research

Biomarkers for evaluating chromium status should be explored in balance studies, where a given amount of chromium is given. Furthermore, long-term effects of increased chromium intake in physiological dosages need to be assessed by clinical trials.

### Conflict of interest and funding

The authors declare no conflicts of interests.
